# ACDA: implementation of an augmented drug synergy prediction algorithm

**DOI:** 10.1093/bioadv/vbad051

**Published:** 2023-04-13

**Authors:** Sergii Domanskyi, Emily L Jocoy, Anuj Srivastava, Carol J Bult

**Affiliations:** The Jackson Laboratory for Mammalian Genetics, Bar Harbor, ME 04609, USA; The Jackson Laboratory, Sacramento, CA 95838, USA; The Jackson Laboratory for Genomic Medicine, Farmington, CT 06032, USA; The Jackson Laboratory for Mammalian Genetics, Bar Harbor, ME 04609, USA

## Abstract

**Motivation:**

Drug synergy prediction is approached with machine learning techniques using molecular and pharmacological data. The published Cancer Drug Atlas (CDA) predicts a synergy outcome in cell-line models from drug target information, gene mutations and the models’ monotherapy drug sensitivity. We observed low performance of the CDA, 0.339, measured by Pearson correlation of predicted versus measured sensitivity on DrugComb datasets.

**Results:**

We augmented the approach CDA by applying a random forest regression and optimization via cross-validation hyper-parameter tuning and named it Augmented CDA (ACDA). We benchmarked the ACDA’s performance, which is 68% higher than that of the CDA when trained and validated on the same dataset spanning 10 tissues. We compared the performance of ACDA to one of the winning methods of the DREAM Drug Combination Prediction Challenge, the performance of which was lower than ACDA in 16 out of 19 cases. We further trained the ACDA on Novartis Institutes for BioMedical Research PDX encyclopedia data and generated sensitivity predictions for PDX models. Finally, we developed a novel approach to visualize synergy-prediction data.

**Availability and implementation:**

The source code is available at https://github.com/TheJacksonLaboratory/drug-synergy and the software package at PyPI.

**Supplementary information:**

Supplementary data are available at *Bioinformatics Advances* online.

## 1 Introduction

Drug combination therapy is a promising approach to cancer treatment (Mokhtari *et al.*, 2017). It is typical that a combination of drugs has no synergy (simultaneously targeting independent pathways) (Palmer and Sorger, 2017). However, a more desirable approach is to use synergistic combinations that increase the therapeutic response rate and have, compared to monotherapies, a lower potential to develop resistance to treatment (Gayvert *et al.*, 2017).

Many computational methods for drug synergy prediction have recently been developed and applied (Wu *et al.*, 2022). A comprehensive review of various methods and their respective input data types was published by Wu *et al.* (2022). The approaches can be categorized into classic machine learning (ML), deep learning and systems biology methods.

A recently developed method, Cancer Drug Atlas, CDA (Narayan *et al.*, 2020), uses drug-target information and monotherapy drug sensitivity for drug-synergy prediction in cell-line models. We developed an in-house implementation of the CDA and re-evaluated it on the Genomics of Drug Sensitivity in Cancer (GDSC) data, demonstrating results consistent with the Narayan *et al.* (2020) publication. However, we observed low performance of the CDA when trained and tested on six datasets from DrugComb (Zheng *et al.*, 2021).

Here, we describe the implementation of improvements to the CDA drug-synergy prediction modeling approach by applying a Classification And Regression Tree (CART)-based model instead of linear regression. Further, we added training optimization and implemented the algorithm as a Python package called Augmented CDA (ACDA). We benchmarked the CDA and ACDA methods on a collection of datasets. Additionally, to support the conclusions of ACDA strengths, we implemented and tested the ACDA alongside a second-best method developed by M. Zaslavskiy (which we denote as EN) of the community-powered DREAM Drug Combination Prediction Challenge to develop and assess computational models for drug-synergy prediction (Menden *et al.*, 2019). The EN method is designed to use one-hot encoded drug and cell line names with ML methods such as Random Forest regression and its variations. We did not use the best method of the DREAM Drug Combination Prediction Challenge, which requires gene expression data for predicting drug effects, as it does not satisfy the ACDA data requirements.

Additionally, we trained and benchmarked ACDA predictions of therapeutic response in Patient-derived Xenograft (PDX) models from the Novartis Institutes for BioMedical Research PDX encyclopedia (NIBR PDXE) collection (Gao *et al.*, 2015) encompassing 281 PDX models of several cancer types. As an example of ACDA application in prioritizing candidate drug combinations for testing in PDX models, we generated a set of model drug–drug candidates for the NIBR PDXE breast cancer subset where drug-combination tumor-volume measurements were unavailable. Finally, we simplified the visualization of drug-sensitivity clustering dendrograms on a 2D layout by using dendrograms and heatmaps instead of the Voronoi diagrams used in the CDA.

## 2 Methods

We formulate the synergy prediction as a supervised ML problem or a regression task to predict a quantitative score. Positive output values indicate synergy between a pair of drugs for a cell line model; negative output values indicate antagonism. The regression output values are predicted from the features of cell lines and drugs.

The Augmented CDA model follows novel aspects compared to those employed by the CDA model (Narayan *et al.*, 2020). We summarized ACDA workflow in Supplementary Figure S1. Step 1 is the same as in the CDA, and Steps 2–4 are modified compared to the CDA:

Step 1. To calculate the drug–drug distance (a drug-similarity measure based on monotherapy sensitivity in the models), first project drug response vectors (n drugs, m cell lines) sensitivity measures (e.g. AUC) to cosine similarity, and construct matrix A. Then calculate Euclidean similarity M (*n* by *n* drugs) of A, hierarchically cluster M with Ward linkage to make a dendrogram. Finally, calculate the cophenetic distance of the clustering.

Step 2. Train a random forest regression model using the monotherapy drug sensitivities, target information (defined as one if any of the two drugs target a gene mutated in a cell line, otherwise zero), and drug–drug distance (i.e. the cophenetic distance derived in Step 1).

Step 3. Compute (predict) synergy values for new or validation data.

Step 4. Visualize predicted and known synergy-pairs data using dendrograms and heatmaps (compare to Voronoi diagrams used in the CDA).

To summarize, in Step 2, random forest regression is used instead of logistic regression, and random search is used for hyperparameter tuning via k-fold cross-validation. In Steps 2–3, compared to a linear model fit evaluation done by Narayan *et al.* (2020), we perform cross-validation using the Monte Carlo Cross-Validation (MCCV) approach, according to which a randomly selected two-third of data was used for training. Then the remaining one-third of the data was used for model performance evaluation (Kuhn and Johnson, 2013). To avoid information leakage, we use stratified splitting to leave out drug combinations. Supplementary Table S1 (excel sheet) shows the results for both stratified and random splitting. The splitting, training, and evaluation were repeated 10 times to benchmark the ACDA and CDA methods. Additional methodological details are provided in the Supplementary Methods.

The EN method uses Random Forest regression and one-hot encoded drug and cell line names. The EN-ACDA method adds ACDA features to the EN method. We finally implemented a method ACDA-EN-ACDA, which averages predictions of ACDA and EN-ACDA.

Similarly to Kuru *et al.* (2022), ACDA ensures that Drug A-Drug B and Drug B-Drug A are used in the training of models. ACDA contains a function that averages the synergy predictions for Drug A-Drug B and Drug B-Drug A to obtain a final synergy score. We reserve the per-pair synergy score averaging function for downstream analysis and do not apply it by default. We use the function on an example data subset described below.

### 2.1 Data description

We used pharmacology and molecular data from (Yang *et al.*, 2013), including GDSC drug sensitivity, model mutations, and drug-targets data (Supplementary Table S2). We manually curated the CDA synergy pairs (Narayan *et al.*, 2020) to harmonize drugs and model names. The GDSC set contains subsets GDSC1 and GDSC2, which are older and newer versions of GDSC. Notably, the GDSC2 subset has been screened using an improved GDSC1 screen design and assay (Yang *et al.*, 2013).

We tested ACDA versus CDA on breast cell lines of various cancer types from the GDSC1 and GDSC2 subsets (the two editions of the GDSC drug screens) with CDA synergy pairs. The GDSC1 breast subset contains 119 drug pairs (54 with synergy and randomly chosen 65 unknown pairs assuming no synergy). The GDSC2 breast subset contains 163 pairs (74 with synergy, 89 without synergy), and GDSC1&2 with 191 pairs (112 with synergy, 79 without synergy). We tested 10 randomly chosen sets of ‘no synergy’ pairs for each scenario.

The second data source was DrugComb which includes 650,909 harmonized drug sensitivity and synergy values from many in vitro studies and tissues (Zheng *et al.*, 2021) (see Supplementary Figs S2 and S3) and, specifically, the AstraZeneca dataset from Menden *et al.* (2019). The data in DrugComb do not have model mutation information; therefore, where available, we used GDSC mutations for DrugComb. We considered only those DrugComb sets for at least 1000 drug–drug-model entries per study per tissue. Because the DrugComb-AstraZeneca dataset is much larger than the GDSC known synergy set, we use the DrugComb subset in a down-sampling experiment using a reduced number of drug–drug-model entries in a training set and a constant number of drug–drug-model entries in the validation set.

As an independent dataset for demonstrating the high benchmarking performance and utility of the ACDA to generate candidate drug–drug combinations in PDX models, we use the NIBR PDXE collection (Gao *et al.*, 2015). The dataset contains tumor-volume response data of 281 models of six cancer types treated with single drugs and drug combinations: colorectal cancer (CRC), gastric cancer (GC), pancreatic ductal adenocarcinoma (PDAC), breast cancer (BRCA), non-small cell lung cancer (NSCLC), and cutaneous melanoma (CM).

### 2.2 Visualization

There are two visualization tools in the ACDA. The first tool allows the clustering of monotherapy sensitivities into a dendrogram of drug–drug similarity and depicts the known synergy pairs as arcs ordered according to the order of the dendrogram leaves. Lighter colors denote a smaller cophenetic distance between drugs, and darker colors show that the drug–drug cophenetic distance is large. The dendrogram is split into 10 clusters. This dendrogram representation allows for visually assessing whether known synergy pairs tend to have large cophenetic distances, as was discussed in the work of Narayan *et al.* (2020). The second tool presents predicted drug-synergy pairs as a clustered heatmap where each point corresponds to a drug pair. Dendrograms reflect the clustering of similarity of drug sensitivities.

### 2.3 Application to PDX models

To analyze PDX model data, we used sensitivity to monotherapy (63 drugs) and tumor mutation profiles of the PDX models from the NIBR PDXE collection (Gao *et al.*, 2015). First, we performed benchmarking via MCCV Scheme repeated 10 times for each of the four methods (ACDA, CDA, EN, and EN-ACDA) and each of the six cancer types listed in Section 2.2, in NIBR PDXE (see Supplementary Methods for methodological details). Next, we used all available drug combinations in the BRCA subset of NIBR PDXE to train the methods and then generated sensitivity predictions for the cases where drug-combination tumor-volume measurements were unavailable. Thus, we calculated and saved the non-averaged synergy scores. We applied the ACDA averaging function, based on the methodology of Kuru *et al.* (2022), to non-averaged BRCA synergy predictions and generated one score for each Drug A–Drug B pair for each method.

## 3 Results

The average Pearson correlation coefficient of predicted versus measured drug-combination sensitivity values and SEM (standard error of the mean) over 10 MCCV iterations for each case of the GDSC and DrugComb-AstraZeneca (DC-AZ) datasets are reported in Table 1. In the case of the GDSC1-breast dataset, the performance of the ACDA was consistently higher or nearly equal to that of the CDA. GDSC and DC-AZ sets were used in the development and validation of CDA. Notably, the CDA correlation coefficient values were similar to those in the CDA publication results (Narayan *et al.*, 2020). However, we showed that the choice of a logistic model reduces the accuracy of drug-response prediction when using the DC-AZ drug screening dataset (and several other GDSC subsets, Table 1). By constructing a CART-based model for the regression and classification problem, we overcame the assumptions necessary for using a logistic model and improved the quality of model performance.

**Table 1.** Benchmark Pearson correlation coefficient of ACDA/CDA models on select tissues and studies

Dataset	Tissue	Train	Test	ACDA	CDA	EN	EN-ACDA	ACDA-EN-ACDA
GDSC1&2	Breast	127	64	0.439 ± 0.080	0.377 ± 0.066	0.394 ± 0.101	0.492 ± 0.083	0.520 ± 0.083
GDSC1	Breast	79	40	0.596 ± 0.032	0.529 ± 0.033	0.700 ± 0.063	0.733 ± 0.032	0.717 ± 0.200
GDSC2	Breast	108	55	0.706 ± 0.054	0.449 ± 0.038	−0.079 ± 0.021	0.594 ± 0.106	0.675 ± 0.064
GDSC1&2	Bladder	73	37	0.501 ± 0.013	0.481 ± 0.037	0.597 ± 0.039	0.656 ± 0.025	0.613 ± 0.015
GDSC1	Bladder	60	30	0.602 ± 0.042	0.634 ± 0.037	0.628 ± 0.040	0.658 ± 0.040	0.647 ± 0.041
GDSC2	Bladder	58	29	0.098 ± 0.083	0.183 ± 0.061	0.454 ± 0.072	0.436 ± 0.061	0.318 ± 0.058
DC-AZ	Breast	6585	3293	0.908 ± 0.006	0.540 ± 0.015^a^	0.278 ± 0.019	0.916 ± 0.005	0.916 ± 0.005
DC-AZ	Lung	2340	1170	0.526 ± 0.017	0.371 ± 0.011^a^	0.382 ± 0.031	0.589 ± 0.018	0.603 ± 0.016
DC-AZ	Urinary tract	2921	1461	0.744 ± 0.021	0.497 ± 0.014^a^	0.331 ± 0.025	0.798 ± 0.017	0.788 ± 0.018

a Linear regression was used instead of logistic regression.

We observed that the performance of the ACDA on a down-sampled DrugComb-AstraZeneca dataset decreased slightly with the smaller training set. In contrast, the performance of the CDA is much lower on the same set. However, the performance of the CDA did not significantly change with the training set size (Supplementary Fig. S4).

We performed validation and benchmarking of our ACDA and CDA implementation using all datasets from DrugComb (Zheng *et al.*, 2021). We compared our model performance of one of the top-performing models, EN, from the DREAM challenge for drug-synergy prediction (Menden *et al.*, 2019). When trained and tested on the same dataset, the EN method shows a performance of 0.414 ± 0.033, comparable to the ACDA’s (0.568 ± 0.041) and the CDA’s (0.339 ± 0.049) performance. However, EN performs poorly (0.009 ± 0.012) when training and testing are done on different studies. In the latter scenario, the ACDA and the CDA have similar but low performances: 0.228 ± 0.046 and 0.215 ± 0.051, respectively. A combination of EN and ACDA is a viable approach (Supplementary Figs S5 and S6), which aligns the strengths of both methods in a generalizable scenario giving 0.63 ± 0.037, or an 86% increase compared to the CDA when trained and tested on subsets of the same study. Averaging predictions of ACDA and EN-ACDA gave a performance of 0.621 ± 0.037.

We additionally designed an experiment to split the GDSC1 data set into 3-1-1 folds, where fine-tuning is done on four folds and validated on the fifth fold while rotating the test fold to reuse data. The hyperparameter tuning gives an 8% performance increase in the tuned model compared to the base model.

The hyperparameter tuning by training on the GDSC2 breast subset and testing on the GDSC1 breast subset led to the increased correlation of the predicted values with ground truth from 0.390 (base model) to 0.797. A similar test using GDSC1 and GDSC2 as a training and testing set suffered from overfitting and decreased correlation from 0.248 (base model) to 0.209.

The results for visualization of the synergistic pairs are detailed in the Supplementary Results, and examples of the two visualization tools are shown in Supplementary Figures S7 and S8.

### 3.1 Application to PDX models

Using MCCV, we applied the ACDA method trained on a randomly selected two-third of measured dual-drug responses stratified to leave out drug combination to predict synergistic combinations (model-specific drug pairs) in NIBR PDXE models (Gao *et al.*, 2015) for which tumor-response values were held out from the training set. The data splitting, training, and model evaluation were repeated 10 times. ACDA, CDA, EN, EN-ACDA and ACDA-EN-ACDA results for each of the six cancer types in NIBR PDXE are detailed in Supplementary Figure S9. ACDA, CDA, and EN-ACDA had similar performances, while EN showed significantly lower performance with GC and PDAC subsets.

Then we used all available drug-combinations data in the breast cancer subset of NIBR PDXE to train the methods and then generated sensitivity predictions for the cases where drug-combination tumor-volume measurements were unavailable, but the computational model predictors were available. The top 30 drug–drug-model synergy candidates with per-pair averaged synergy scores are listed in Supplementary Table S3, with model X-4567 appearing in the top 30. A complete list of predicted sensitivity to drug combinations predicted from ACDA, CDA, EN and EN-ACDA for each model is provided in Supplementary Table S4, which contains both averaged and non-averaged scores. The table contains computational predictions for 39 breast cancer PDX models and 119 unique drug–drug combinations sorted by EN-ACDA score in descending order. Several drug combinations, including ribociclib (LEE011) combined with paclitaxel, LLM871, infigratinib (BGJ398), binimetinib, buparlisib (BKM120) and CLR457, are predicted to have a synergistic effect in many of the 39 models. Red squares on the heatmap in Supplementary Figure S10 denote such drug combinations. Supplementary Table S5 contains the heatmap values and drug names from Supplementary Figure S10. Additionally, ACDA, CDA, EN and EN-ACDA give consistent results in this calculation. For example, a synergy score of 0.75 corresponds to CR in the mapping of response categories to synergy scores in Supplementary Methods. The overlap in drug–drug-model entries for which the predicted synergy score is at least 0.75 is significant with hypergeometric test *P*-value <$10^{-31}$ when comparing all pairs of the four methods.

## 4 Conclusion

The newly developed ACDA method is an open-source software package and includes the CDA, EN, and EN-ACDA implementations. Benchmarking the four methods demonstrated that ACDA and EN-ACDA were better at predicting synergistic drug combinations than CDA and EN. The ACDA approach can be used with cell-line drug screens and *in vivo* PDX tumor-volume data to predict drug–drug combinations that may synergistically affect specific models.

## Supplementary Material

vbad051_Supplementary_Data

## Data Availability

All the data collections, including the DrugComb, GDSC and NIBR PDXE datasets, are publicly available. The ACDA source code, documentation, examples of usage and example data are openly available at https://github.com/TheJacksonLaboratory/drug-synergy and https://acda.readthedocs.io/. The list of curated CDA synergy pairs can be found in Supplementary Table S6.
