# Supplementary material for: ACDA: implementation of an augmented drug synergy prediction algorithm
Source: Bioinform Adv. 2023 Apr 13;3(1):vbad051. doi: 10.1093/bioadv/vbad051 (PMC10125903; doi:10.1093/bioadv/vbad051)
Supplement: vbad051_Supplementary_Data [file vbad051_supplementary_data.zip › Drug_Synergy_supp_oxford.docx]

ACDA: Implementation of an Augmented Drug Synergy Prediction Algorithm

Sergii Domanskyi^1^, Emily L. Jocoy^3^, Anuj Srivastava^2*^, and Carol J. Bult^1*^

^1^The Jackson Laboratory for Mammalian Genetics, Bar Harbor, 04609, USA, ^2^The Jackson Laboratory for Genomic Medicine, Farmington, Connecticut, 06032, USA, ^3^The Jackson Laboratory, Sacramento, California, 95838, USA.

*To whom correspondence should be addressed.

Supplementary Information

# Supplementary Methods

## Machine Learning

Due to a small number of known synergy pairs (approximately 100 on average for GDSC breast cancer cell lines) in the curated CDA dataset, our experimental design is based on the stratified (to leave drug combinations out (Preuer et al. 2018)) Monte Carlo Cross-Validation scheme. We reported average performance over ten training iterations on two-thirds of randomly sampled data and tested the model on the remaining one-third.

We also perform independent validation with model training on the GDSC2 breast cancer subset and validation of the model on the GDSC1 breast cancer subset. In this scenario, we show how ACDA hyperparameter tuning applied to the GDSC2 subset improves the performance on the validation GDSC1 subset compared to an untuned base model.

Similarly, we perform a set of experiments across a range of datasets from DrugComb by training on data from one study and testing on a different study. Specifically, we randomly select one-half of the data from one study for training and validate the model on randomly selected one-half of the data from the second study. Training and validation are repeated ten times. We report average performance and error.

All the implemented methods we developed or used do not guarantee identical drug synergy prediction for Drug A-Drug B as the Linear, Logistic, and Random Forest Regressions do not constrain the two CDA sensitivity covariates to have equal weight. Similarly, the weights of covariates derived from the one-hot encoded drug and cell line names are not constrained.

## Drug synergy scoring with MuSyC

Multi-dimensional Synergy of Combinations (MuSyc) (Wooten et al. 2021) is a robust mathematical framework and a generalization formalism for the calculation of drug synergy. MuSyC generalizes many synergy metrics, such as Bliss, Loewe, CI, HSA, Effective Dose model, ZIP, Hill PDE, and GPDI. The algorithm reconstructs the 2D drug synergy response from two 1D drug response vectors, i.e., raw drug sensitivity data.

**Supplementary Table 2.** **Description of the datasets used in this work.** The sources of the data used in our work are DrugComb (Zheng et al. 2021), GDSC (Yang et al. 2013), and CDA (Narayan et al. 2020). The PDX resource used in our analysis is NIBR PDXE (Gao et al. 2015).

| Data name | Number of models | Number of drugs | Number of drug-model entries (monotherapy experiments) | Number of drug-drug-model entries (drug combination experiments) | Resource reference |
| --- | --- | --- | --- | --- | --- |
| DrugComb collection of 26 studies* | 1995 | 4622 | 1306771 | 650909 | https://drugcomb.fimm.fi/download/ |
| GDSC1 Drug screen | 987 | 345 | 292849 | 0 | https://www.cancerrxgene.org/downloads/bulk_download |
| GDSC2 Drug screen | 809 | 192 | 131108 | 0 | https://www.cancerrxgene.org/downloads/bulk_download |
| CDA synergy | 109 | 240 | 0 | 437 | <https://doi.org/10.1038/s41467-020-16735-2>, Supplementary Data 2 |
| GDSC-Sanger cell line models mutations | 1032 | n/a | n/a | n/a | https://cellmodelpassports.sanger.ac.uk/downloads |
| GDSC-Sanger drug targets | n/a | 442 | n/a | n/a | https://www.cancerrxgene.org/downloads/bulk_download |
| NIBR PDXE | 281 | 63 | 3479 | 1237 | <https://www.nature.com/articles/nm.3954>, Supplementary Table 1 |

*Includes AstraZeneca study

The DrugComb dataset (Zheng et al. 2021) contains all measured drug pairs regardless of the synergy value. In GDSC, drug-synergy pairs have binary “synergy, no synergy” values; therefore, we applied the CDA with linear regression instead of logistic regression.

Since the ACDA is based on several features from different data types (pharmacology and molecular), data curation is crucial. The underlying software uses models, drugs, and gene identifiers to match and query the data tables, and these identifiers were curated to be consistent across the tables. Curated data is shared for reproducibility, and documentation is available at <https://acda.readthedocs.io>.

Hyperparameter optimization was performed by a randomized search with k-fold cross-validation (k=3) using one data subset as a training dataset. The best estimator was determined by performance on another subset.

## Application to PDX models

In order to apply ACDA to PDX data from NIBR PDXE, the steps outlined below must be carried out:

1. Select the PDX models of one cancer type (e.g., BRCA).
2. Generate ACDA-formatted data by following the instructions at <https://acda.readthedocs.io/en/latest/data.html#alternative-data-format-simple> and <https://acda.readthedocs.io/en/latest/examples.html#examples>.
3. Map the tumor volume-derived response category to numeric values, i.e., CR: 1, PR: 0.5, SD: -0.25, PD: -1, SD->PD: -0.75, SD-->-->PD: -0.5, CR-->PD: 0.75, CR-->-->PD: 0.75, PR-->PD: 0.25, PR-->-->PD: 0.25.
4. Train a classifier on drug-drug model entries with no missing features for which response is known.
5. Generate response predictions on drug-drug-model entries with no missing features for which the response is not known.

# Supplementary Results

## Visualization of the synergistic pairs

The visualization example of clustering drug-sensitivity measure values overlaid with known drug-synergy values for the DrugComb-AstraZeneca breast cancer subset is shown in Supplementary Figure 7. This dendrogram representation with color-coded arcs allows researchers to determine that known synergy pairs tend to have large cophenetic distances. Another visualization example is a heatmap of the synergy-pairs predictions for the GDSC2 breast subset, where each point on the heatmap corresponds to a drug pair. The dendrograms reflect the clustering of the similarity of drug sensitivities (Supplementary Figure 8).

**Supplementary Table 3.** **Top drug-synergy combination candidates among 39 breast cancer PDX models and 119 unique drug-drug combinations sorted by EN-ACDA score.** By using EN-ACDA, we train a classifier on PDX models for measured drug combinations and predict synergy for PDX models where the drug-combination response is unknown. The top 20 synergy-pairs candidates are listed. A complete list of 9184 predicted sensitivity to drug-drug combinations for each model is shown in Supplementary Table 4.

| Model | Drug 1 | Drug 2 | ACDA | CDA | EN | EN-ACDA |
| --- | --- | --- | --- | --- | --- | --- |
| X-4567 | BYL719 | LLM871 | 0.917583333 | 1.999955812 | 0.939 | 0.94 |
| X-4567 | LLM871 | BYL719 | 0.917583333 | 1.999955812 | 0.939 | 0.94 |
| X-4567 | paclitaxel | BYL719 | 0.917583333 | 1.999955812 | 0.939 | 0.94 |
| X-4567 | BYL719 | paclitaxel | 0.917583333 | 1.999955812 | 0.939 | 0.94 |
| X-4567 | binimetinib | BYL719 | 0.654691667 | 1.629797247 | 0.939 | 0.9305 |
| X-4567 | BYL719 | binimetinib | 0.654691667 | 1.629797247 | 0.939 | 0.9305 |
| X-4567 | LLM871 | LJM716 | 0.710695635 | 0.944511298 | 0.985 | 0.9285 |
| X-4567 | paclitaxel | LJM716 | 0.710695635 | 0.944511298 | 0.985 | 0.9285 |
| X-4567 | LJM716 | BKM120 | 0.710695635 | 0.921354499 | 0.985 | 0.9285 |
| X-4567 | LJM716 | paclitaxel | 0.710695635 | 0.944511298 | 0.985 | 0.9285 |
| X-4567 | BKM120 | LJM716 | 0.710695635 | 0.921354499 | 0.985 | 0.9285 |
| X-4567 | LJM716 | CLR457 | 0.710695635 | 0.921354499 | 0.985 | 0.9285 |
| X-4567 | LJM716 | LLM871 | 0.710695635 | 0.944511298 | 0.985 | 0.9285 |
| X-4567 | CLR457 | LJM716 | 0.710695635 | 0.921354499 | 0.985 | 0.9285 |
| X-4567 | LLM871 | CLR457 | 0.917583333 | 1.999955812 | 0.938 | 0.926 |
| X-4567 | BKM120 | paclitaxel | 0.917583333 | 1.999955812 | 0.938 | 0.926 |
| X-4567 | LLM871 | BKM120 | 0.917583333 | 1.999955812 | 0.938 | 0.926 |
| X-4567 | CLR457 | LLM871 | 0.917583333 | 1.999955812 | 0.938 | 0.926 |
| X-4567 | BKM120 | LLM871 | 0.917583333 | 1.999955812 | 0.938 | 0.926 |
| X-4567 | paclitaxel | BKM120 | 0.917583333 | 1.999955812 | 0.938 | 0.926 |
| X-4567 | paclitaxel | CLR457 | 0.917583333 | 1.999955812 | 0.938 | 0.926 |
| X-4567 | CLR457 | paclitaxel | 0.917583333 | 1.999955812 | 0.938 | 0.926 |
| X-4567 | BYL719 | CLR457 | 0.882583333 | 1.953854264 | 0.939 | 0.924 |
| X-4567 | BKM120 | BYL719 | 0.882583333 | 1.956382828 | 0.939 | 0.924 |
| X-4567 | BYL719 | BKM120 | 0.882583333 | 1.956382828 | 0.939 | 0.924 |
| X-4567 | CLR457 | BYL719 | 0.882583333 | 1.953854264 | 0.939 | 0.924 |
| X-4567 | binimetinib | LLM871 | 0.654691667 | 1.648140974 | 0.938 | 0.9155 |
| X-4567 | paclitaxel | binimetinib | 0.654691667 | 1.648140974 | 0.938 | 0.9155 |
| X-4567 | BKM120 | binimetinib | 0.654691667 | 1.629797247 | 0.938 | 0.9155 |
| X-4567 | binimetinib | CLR457 | 0.654691667 | 1.629797247 | 0.938 | 0.9155 |

## Future Directions

The current ACDA synergy predictions are partly dependent on the experimental system model (PDX or cell line) gene mutations. Future versions of ACDA will include genes downstream of the drug targets using known protein-protein interaction networks known as parsimonious composite networks (Huang et al. 2018). Expanding the gene lists to include nearest neighbor genes based on protein-protein interaction networks will also be evaluated to see if the performance of ACDA can be enhanced. Finally, future studies will integrate into ACDA information about the overlap of mutations with sets of ontology pathways to define a new covariate in the regression. For example, PDX model X-4567 is predicted to be sensitive to paclitaxel and binimetinib (Supplementary Table 3). This model has mutations in nine of the 267 MAPK pathway-related genes (Subramanian et al. 2005). The combination of paclitaxel and binimetinib was studied in clinical trial NCT01649336 and was shown to have a clinical benefit (57% increase in CR+PR+SD) in treating epithelial ovarian tumors harboring alterations in the MAPK pathway (Grisham et al. 2018). Thus, adding pathway context to the model may further enhance the drug synergy predictions.

# Supplementary Figures


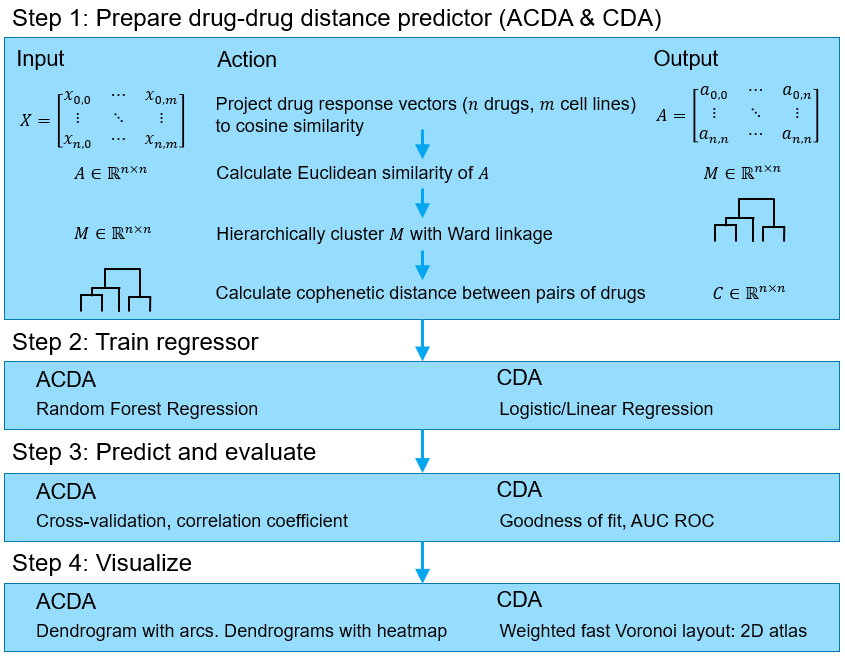


**Figure S1. ACDA workflow.** Step 1 is identical in ACDA and CDA. Steps 2-4 shows comparison of ACDA to CDA. AUC ROC is area under the receiver operating characteristic curve.


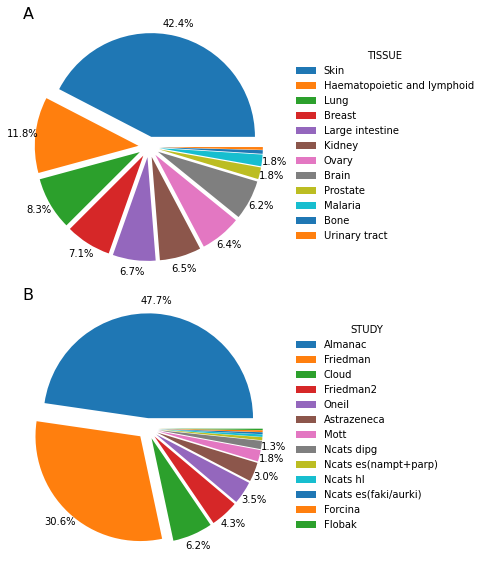


**Figure S2.** **DrugComb data composition overview by tissues and by studies.** Distribution of drug pairs in DrugComb (Zheng et al. 2021), a comprehensive drug-sensitivity data repository and analysis portal, separated by (A) studies and (B) tissues. Only those study-tissue cases with at least 1,000 measured drug pairs are shown. Overall, DrugComb collects more than 6500,00 model-drug-drug entries, spanning 26 studies, 288 cell-line models, and 4,268 drugs. Skin is the best-represented tissue in this data resource, accounting for 42.4% of all data entries. Similarly, the “Almanac” and “Friedman” studies account for 78.3% of the data; however, other valuable studies such as “AstraZeneca” and “Oneil” are included as well.

**
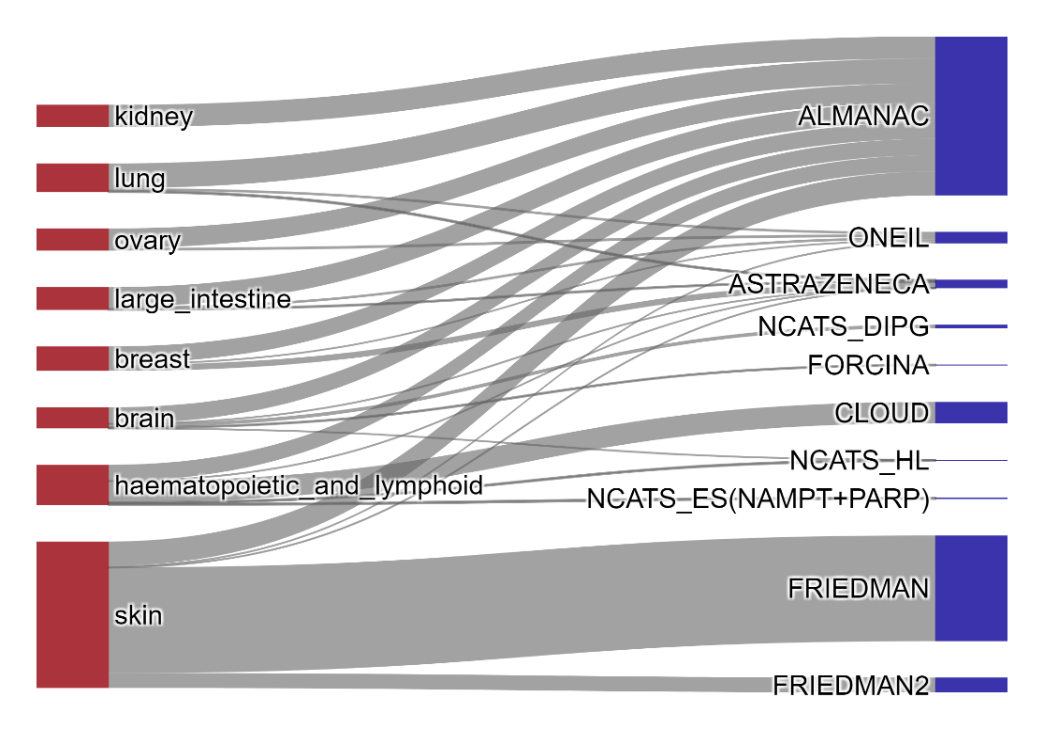
**

**Figure S3. Comparison of the tissues and studies from DrugComb highlighting the weight of each study by the data quantity.** DrugComb collects measured drug pairs from several tissues and studies. Only those study-tissue cases that have at least 1,000 measured drug pairs are shown. Noticeably, a significant fraction of all pairs is from skin. The largest studies in this dataset are “Almanac” and “Friedman” (Zheng et al. 2021). The figure was generated with the visualization tools of Digital Cell Sorter (Domanskyi et al. 2019, 2021).


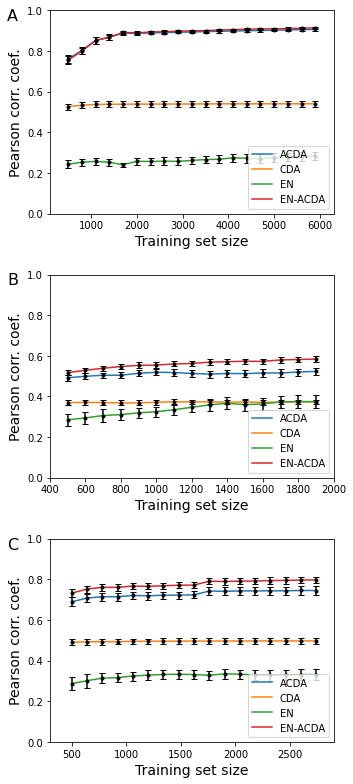


**Figure S4.** **Effect of down-sampling for DrugComb AstraZeneca training dataset.** The panels correspond to three tissues from the AstraZeneca dataset and contain (A) 9,878 entries in the breast, (B) 3,510 entries in the lung, and (C) 4,382 entries in the urinary tract set. Training is done on a subsample of 2/3 of the randomly split data, and testing is done on 1/3 of the data. For each training set size, random split and subsampling are repeated 10 times. The random splits are reused across all training set sizes. A consistent effect is observed: (i) ACDA performance is higher than that of CDA, while both ACDA/CDA have nearly constant performance across a range of training set sizes, (ii) EN performance is significantly lower than that of the other methods in (A) and (C), (iii) Addition of EN features to ACDA (see EN-ACDA labeled curves) improves ACDA performance.

**
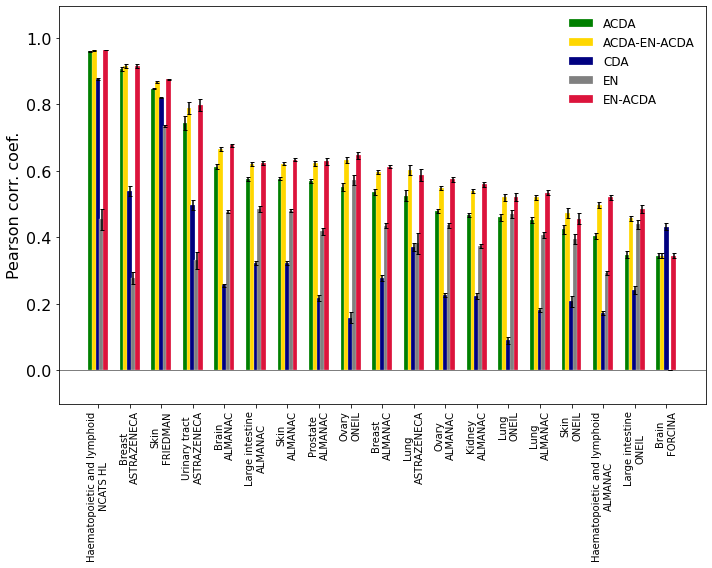
**

**Figure S5.** **Comparison of drug-synergy prediction methods across a range of datasets from DrugComb by training and testing on data within the same studies.** Benchmarking the synergy-prediction methods on DrugComb datasets for which there are at least 1,000 entries per tissue and study after any entries with missing values were removed. Model mutations data are taken from the GDSC Sanger dataset where available. Training is done on 2/3 of the data and testing on 1/3 of the data of the same dataset, split in a stratified scheme to leave drug combinations out, and repeated over 10 splits in a Monte Carlo Cross-Validation scheme. The chart shows Pearson correlation coefficients of the experimentally measured synergy values with values predicted by the four methods. Black error bars show SEM over 10 Monte Carlo iterations. ACDA performance is consistently higher than that of CDA for most of the tested datasets. Using EN features with a random forest regression (EN-labelled curves) leads to the highest performance for most of the datasets. The EN-ACDA combination shows the highest performance across nearly all tested datasets.


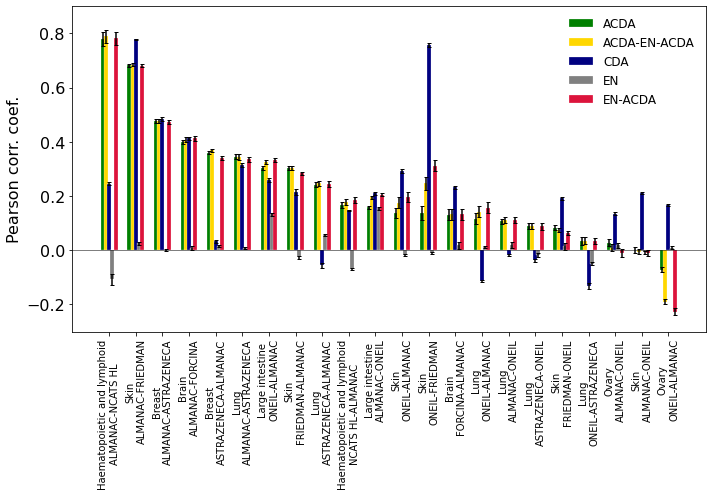


**Figure S6.** **Comparison of drug-synergy prediction methods across a range of datasets from DrugComb by training on data from one study and testing on a different study.** Benchmarking synergy prediction methods on DrugComb datasets for which there are at least 1,000 entries per tissue and study after any entries with missing values were removed. Note that for model mutations, data is taken from the GDSC Sanger dataset where available. Training is done on 1/2 of the data from one study, testing on 1/2 of the data of another study specified in the figure labels, repeated over 10 random splits in a Monte Carlo Cross-Validation-like scheme. The chart shows Pearson correlation coefficients of the experimentally measured synergy values with values predicted by the four methods. Black error bars show SEM over 10 Monte Carlo iterations. ACDA, CDA, EN-ACDA, and ACDA-EN-ACDA have similar performance. Because the datasets share limited overlap, the EN approach alone gives near zero correlation with ground truth values; therefore, it cannot be used when training and testing data do not overlap in models and drugs.


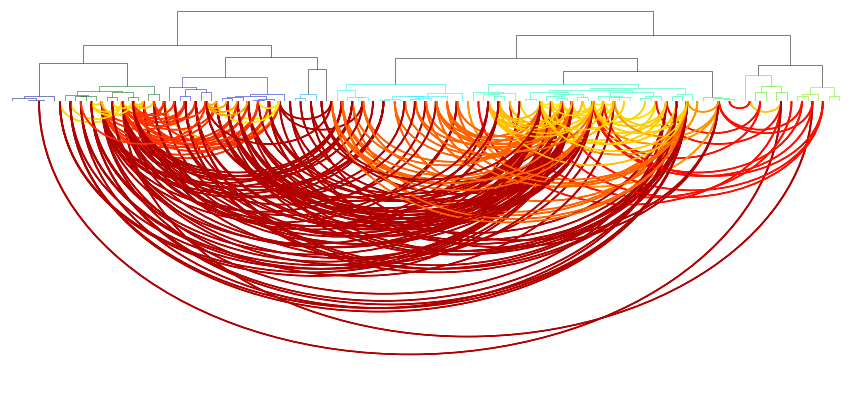


**Figure S7**. **Clustering of drug-sensitivity measure values overlaid with known drug-synergy values.** DrugComb AstraZeneca breast cancer cell-line sensitivity (AUC) is clustered into a dendrogram of drug-drug similarity (top panel), and visualization of the known synergy pairs (bottom panel) is ordered according to the order of the dendrogram leaves. The pairs with a synergy score of at least 20 are assumed to be known synergy pairs. Lighter colors (yellow) denote a smaller cophenetic distance between drugs, and darker colors show that the drug-drug cophenetic distance is large. The dendrogram is split into 10 clusters and color-coded. This dendrogram representation allows visual assessment of whether known synergy pairs tend to have large cophenetic distances, as was discussed in the work of (Narayan et al. 2020).


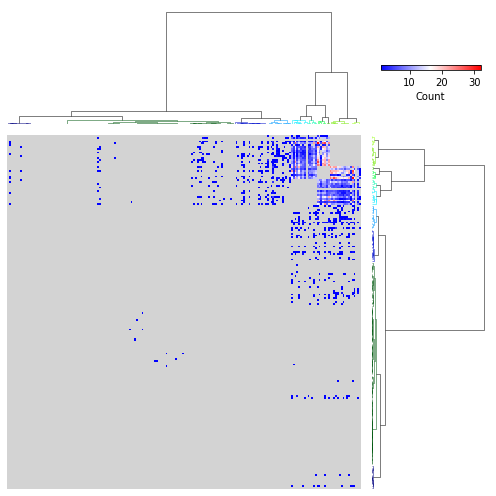


**Figure S8**. **Heatmap of the synergy-pairs predictions for GDSC2 breast subset.** Each point on the heatmap corresponds to a drug pair. Dendrograms reflect the clustering of similarity of drug sensitivities. The color reflects the number of cell-line models where the drug combination may have a synergistic effect, with regression values of at least 0.95. We observe that the top right area of the heatmap tends to contain many synergistic combinations, i.e., a small subset of drugs from the top right section of the dendrogram is predicted to have many synergistic pairs among them.


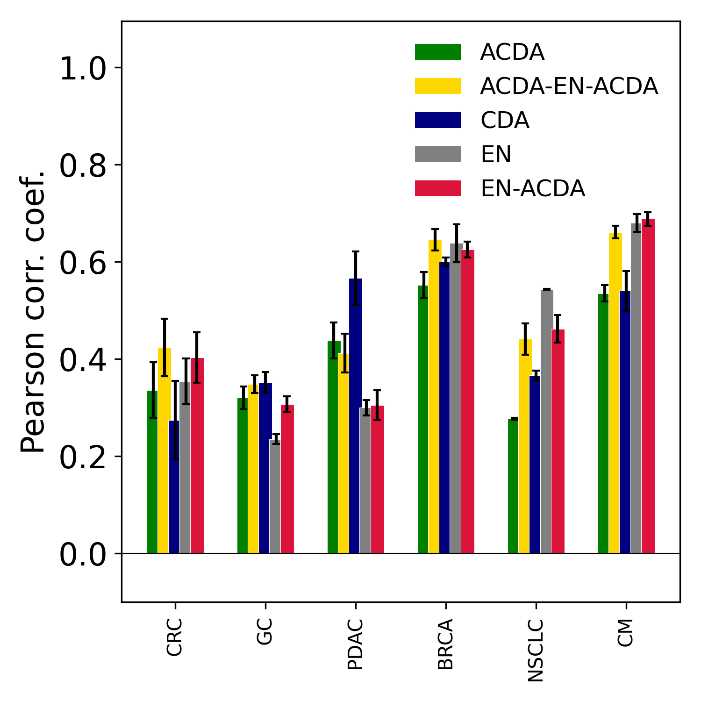


**Figure S9.** **Comparison of drug-synergy prediction methods across a range of cancer types from the NIBR PDXE dataset.** Benchmarking synergy prediction methods on PDX models of six DrugComb datasets (one dataset for each of six cancer types) for which monotherapy and mutation profiles are available. There are at least 117 drug-drug model entries with non-missing features for each cancer type (CRC: 294, GC: 270, PDAC: 219, BRCA: 188, NSCLC: 148, CM: 117). For each cancer type, training is done on 2/3 of the data, and testing on 1/3 of the data from the same cancer type, is repeated over 10 random splits in a Monte Carlo Cross-Validation-like scheme. The chart shows Pearson correlation coefficients of the experimentally measured synergy values with values predicted by the four methods. Black error bars show SEM over 10 Monte Carlo iterations. ACDA, CDA, and EN-ACDA have similar performances, while EN shows significantly lower performance with the GC and PDAC subsets. The average predictions of ACDA and EN-ACDA show good performance in all six cancer types.


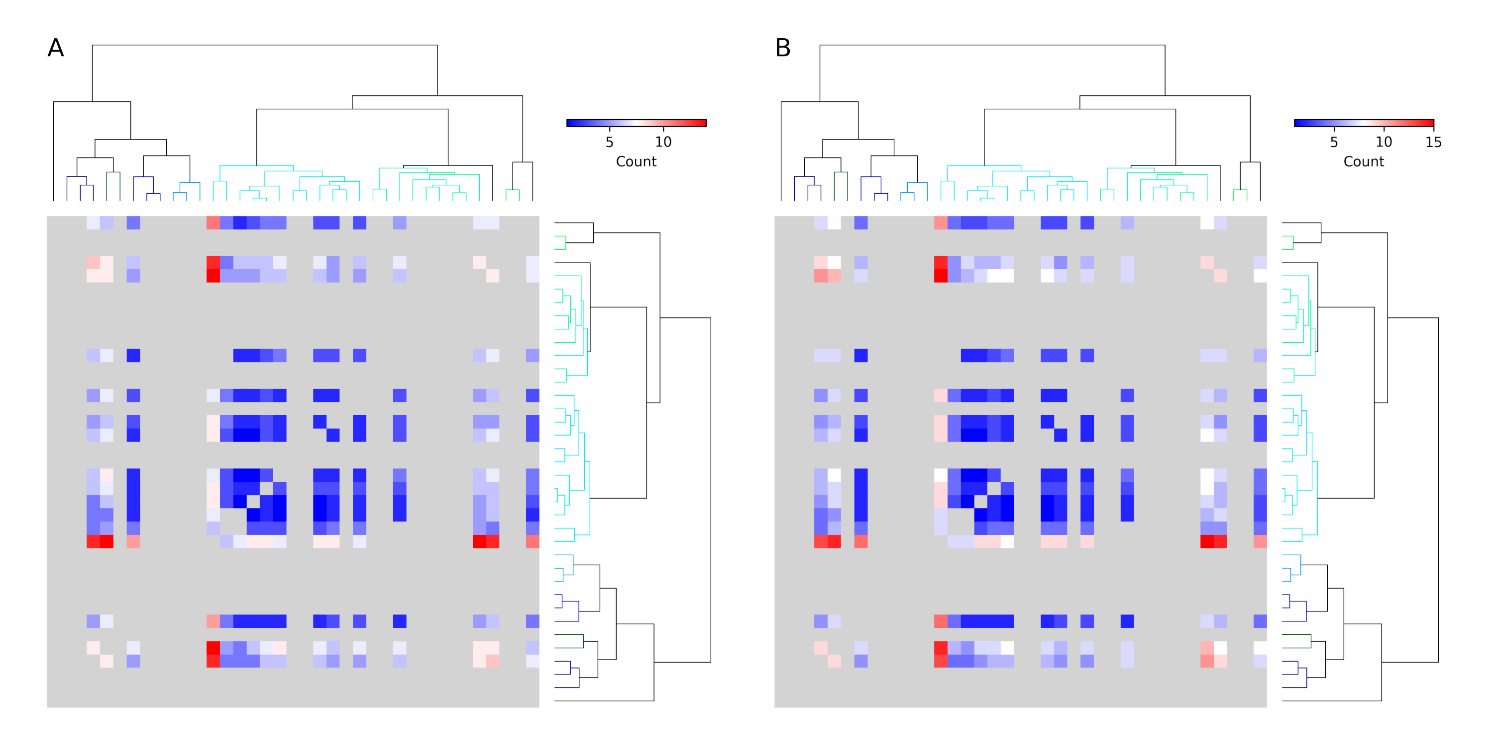


**Figure S10**. **Heatmap of the synergy-pairs predictions by EN-ACDA for NIBR PDXE BRCA subset.** (A) Averaged, (B) Non-averaged synergy scores. Each point on the heatmaps corresponds to a drug pair. Dendrograms reflect the clustering of similarity of drug sensitivities. The color reflects the number of cell-line models in which the drug combination may have a synergistic effect, with predicted scores of at least 0. We observe that a subset of drugs is predicted to have a synergistic effect in many models.

Supplementary References

Bansal, M., Yang, J., Karan, C., Menden, M. P., Costello, J. C., Tang, H., Xiao, G., et al. (2014). ‘A community computational challenge to predict the activity of pairs of compounds’, *Nature Biotechnology*, 32/12: 1213–22.

Baptista, D., Ferreira, P. G., & Rocha, M. (2022). ‘A systematic evaluation of deep learning methods for the prediction of drug synergy in cancer’, *PLOS Computational Biology*. https://doi.org/10.1371/journal.pcbi.1010200.

Domanskyi, S., Hakansson, A., Bertus, T. J., Paternostro, G., & Piermarocchi, C. (2021). ‘Digital Cell Sorter (DCS): a cell type identification, anomaly detection, and Hopfield landscapes toolkit for single-cell transcriptomics’, *PeerJ*, 9: e10670.

Domanskyi, S., Szedlak, A., Hawkins, N. T., Wang, J., Paternostro, G., & Piermarocchi, C. (2019). ‘Polled Digital Cell Sorter (p-DCS): Automatic identification of hematological cell types from single cell RNA-sequencing clusters’, *BMC bioinformatics*, 20/1: 369.

Gao, H., Korn, J. M., Ferretti, S., Monahan, J. E., Wang, Y., Singh, M., Zhang, C., et al. (2015). ‘High-throughput screening using patient-derived tumor xenografts to predict clinical trial drug response’, *Nature Medicine*, 21/11: 1318–25. Nature Publishing Group.

Grisham, R. N., Moore, K. N., Gordon, M. S., Harb, W., Cody, G., Halpenny, D. F., Makker, V., et al. (2018). ‘Phase Ib Study of Binimetinib with Paclitaxel in Patients with Platinum-Resistant Ovarian Cancer: Final Results, Potential Biomarkers, and Extreme Responders’, *Clinical cancer research : an official journal of the American Association for Cancer Research*, 24/22: 5525–33.

Huang, J. K., Carlin, D. E., Yu, M. K., Zhang, W., Kreisberg, J. F., Tamayo, P., & Ideker, T. (2018). ‘Systematic Evaluation of Molecular Networks for Discovery of Disease Genes’, *Cell Systems*, 6/4: 484-495.e5. Elsevier.

Nam, J., Son, S., Park, K. S., Zou, W., Shea, L. D., & Moon, J. J. (2019). ‘Cancer nanomedicine for combination cancer immunotherapy’, *Nature Reviews Materials*, 4/6: 398–414. Nature Publishing Group.

Narayan, R. S., Molenaar, P., Teng, J., Cornelissen, F. M. G., Roelofs, I., Menezes, R., Dik, R., et al. (2020). ‘A cancer drug atlas enables synergistic targeting of independent drug vulnerabilities’, *Nature Communications*, 11/1: 2935. Nature Publishing Group.

Patel, S. A., & Minn, A. J. (2018). ‘Combination Cancer Therapy with Immune Checkpoint Blockade: Mechanisms and Strategies’, *Immunity*, 48/3: 417–33.

K. Preuer, R. P. I. Lewis, S. Hochreiter, A. Bender, K. C. Bulusu, G. Klambauer (2018). DeepSynergy: predicting anti-cancer drug synergy with Deep Learning. *Bioinformatics* (Oxford, England), 34/9: 1538–1546.

Subramanian, A., Tamayo, P., Mootha, V. K., Mukherjee, S., Ebert, B. L., Gillette, M. A., Paulovich, A., et al. (2005). ‘Gene set enrichment analysis: A knowledge-based approach for interpreting genome-wide expression profiles’, *Proceedings of the National Academy of Sciences*, 102/43: 15545–50. Proceedings of the National Academy of Sciences.

Sun, W., Sanderson, P., & Zheng, W. (2016). ‘Drug combination therapy increases successful drug repositioning’, *Drug discovery today*, 21/7: 1189–95.

Torkamannia, A., Omidi, Y., & Ferdousi, R. (2022). ‘A review of machine learning approaches for drug synergy prediction in cancer’, *Briefings in Bioinformatics*, bbac075.

Wu, L., Wen, Y., Leng, D., Zhang, Q., Dai, C., Wang, Z., Liu, Z., et al. (2022). ‘Machine learning methods, databases and tools for drug combination prediction’, *Briefings in Bioinformatics*, 23/1: bbab355.

Yang, W., Soares, J., Greninger, P., Edelman, E. J., Lightfoot, H., Forbes, S., Bindal, N., et al. (2013). ‘Genomics of Drug Sensitivity in Cancer (GDSC): a resource for therapeutic biomarker discovery in cancer cells’, *Nucleic Acids Research*, 41/Database issue: D955-961.

Zheng, S., Aldahdooh, J., Shadbahr, T., Wang, Y., Aldahdooh, D., Bao, J., Wang, W., et al. (2021). ‘DrugComb update: a more comprehensive drug sensitivity data repository and analysis portal’, *Nucleic Acids Research*, 49/W1: W174–84.

D. J. Wooten, C. T. Meyer, A. L. R. Lubbock, V. Quaranta, C. F. Lopez (2021). ‘MuSyC is a consensus framework that unifies multi-drug synergy metrics for combinatorial drug discovery’, *Nature Communications*, 12: 4607.
